# Kids in the city study: research design and methodology

**DOI:** 10.1186/1471-2458-11-587

**Published:** 2011-07-24

**Authors:** Melody Oliver, Karen Witten, Robin A Kearns, Suzanne Mavoa, Hannah M Badland, Penelope Carroll, Chelsea Drumheller, Nicola Tavae, Lanuola Asiasiga, Su Jelley, Hector Kaiwai, Simon Opit, En-Yi Judy Lin, Paul Sweetsur, Helen Moewaka Barnes, Nic Mason, Christina Ergler

**Affiliations:** 1 Centre for Physical Activity and Nutrition, Auckland University of Technology, Auckland, NZ; 2 SHORE and Whariki Research Centre, School of Public Health, Massey University, Auckland, NZ; 3 School of Environment, The University of Auckland, Auckland, NZ; 4 Department of Epidemiology and Public Health, Division of Population Health, University College London, London, UK; 5 Institute of Public Policy, Auckland University of Technology, Auckland, NZ

## Abstract

**Background:**

Physical activity is essential for optimal physical and psychological health but substantial declines in children's activity levels have occurred in New Zealand and internationally. Children's independent mobility (i.e., outdoor play and traveling to destinations unsupervised), an integral component of physical activity in childhood, has also declined radically in recent decades. Safety-conscious parenting practices, car reliance and auto-centric urban design have converged to produce children living increasingly sedentary lives. This research investigates how urban neighborhood environments can support or enable or restrict children's independent mobility, thereby influencing physical activity accumulation and participation in daily life.

**Methods/Design:**

The study is located in six Auckland, New Zealand neighborhoods, diverse in terms of urban design attributes, particularly residential density. Participants comprise 160 children aged 9-11 years and their parents/caregivers. Objective measures (global positioning systems, accelerometers, geographical information systems, observational audits) assessed children's independent mobility and physical activity, neighborhood infrastructure, and streetscape attributes. Parent and child neighborhood perceptions and experiences were assessed using qualitative research methods.

**Discussion:**

This study is one of the first internationally to examine the association of specific urban design attributes with child independent mobility. Using robust, appropriate, and best practice objective measures, this study provides robust epidemiological information regarding the relationships between the built environment and health outcomes for this population.

## Background

Physical activity participation in childhood is essential for optimal physical and psychological health. It is associated with improved bone density, blood lipid and lipoprotein profiles, glucose metabolism, reduced adiposity and blood pressure [1,2] and lower levels of body fat mass in later life [3]. Despite these associations, children's physical activity accumulation has decreased in New Zealand as in many developed countries [4,5]. Independent mobility (i.e., outdoor play and traveling to destinations unsupervised), an integral component of physical activity accumulation in childhood, has also declined radically in recent decades [6-9]. In particular, substantial declines have occurred in children's walking for transport, including the trip to school [10].

Safety-conscious parenting practices, car reliance and auto-centric urban design have converged to produce children living increasingly sedentary lives, chauffeured between activities and under adult surveillance while outdoors [6,11]. In addition, parents chaperoning children in cars creates a 'social trap', whereby fewer children and more cars on the streets increase risks for remaining pedestrians and cyclists, child or otherwise [12-14]. The absence of children in public spaces also diminishes the sense of place and social cohesion experienced by others [15]. Independent mobility also has an important function in childhood development; children learn through autonomous interaction with their people and place surroundings [16] and experiences of place contribute to self-identity, security, and social competence [17]. Restricting play and independent mobility opportunities may curb children's interactions with others and the environment, thereby impacting on their social, emotional, cognitive, and physical development [18,19].

Understanding and modifying the contextual environment in which activity occurs (e.g., active travel), as part of an ecological framework, offers a sustainable solution to increasing physical activity and independent mobility for improved health [20,21]. While numerous urban design characteristics (e.g., residential density, walkability) are advocated for sustainable changes to adult physical activity and travel behaviors [22], little is known of the association between the urban form and children's physical activity, particularly independent mobility behaviors, with inconsistent findings being reported [23]. Differences in independent mobility for children have been shown by age, sex, setting, and socioeconomic status, and differential effects of the urban environment on physical activity and independent mobility have been found by characteristics of the journey (e.g., destination, distance). Older children are more likely to roam further than younger children, and those residing in neighborhoods with greater street connectivity are more active outside their back/front yard when compared with children who live in cul-de-sac networks [24]. Conflicting results have been found with respect to residential density, with a positive relationship found for children's walking and cycling trip lengths [25], and a negative association for children's mobility in high residential density areas (i.e., multi-story apartment blocks) compared with those living in suburban and rural areas [26]. 'Vertical' living children in multi-storied public housing estates in inner city Melbourne, Australia, had more limited play geographies and travel range than children from privately owned apartments, highlighting potentially important neighborhood-level socioeconomic influences [9]. With respect to sex, boys tend to be granted more freedoms than girls, whereas girls negotiate collective independence [27]. Neighborhood environment features such as amenity access, green space, visible incivilities, traffic volumes and pedestrian infrastructure may influence the independent mobility of both sexes [16,28]. Surveillance of outdoor spaces from apartments has also been associated with parents affording children greater license to venture outdoors without supervision [29]. From a young person's perspective, children may favor public spaces where others of a similar age congregate [9].

The importance of children's independent mobility has been established from a social perspective and health associations have been drawn with children's active travel and physical activity. Yet, little is known regarding how specific built environmental factors at the neighborhood level contribute to facilitating independent mobility and physical activity in children, and how children's and parents' environmental perceptions and experiences influence children's objectively assessed activity and independent mobility. Notwithstanding this lack of knowledge, urban intensification policies are being advocated in New Zealand cities and elsewhere, causing neighborhoods to change in ways that may have profound impacts on children's future wellbeing. Understanding these relationships more fully will enable child health outcomes to be considered alongside adult needs in urban neighborhood policy and planning. Accordingly, this study seeks to build upon the existing evidence base by investigating these relationships using current best practice objective approaches to inform the building of healthy cities for continued and sustainable promotion of physical activity behaviors in children.

### Study aims

The 'Kids in the City' (**KITC**) study seeks to understand how the physical design and residential density of urban neighborhoods in higher deprivation areas can influence the physical activity and independent mobility in resident children aged 9-11 years. Specific objectives were as follows:

1. To understand children's perceptions and experiences of neighborhood spaces and the opportunities and constraints they face moving between settings and activities of daily life.

2. To investigate how child and parent safety-versus-independence discourses and mobility-related practices reflect neighbourhood environmental factors (physical and social).

3. To investigate the tensions and margins for change revealed in these discourses and practices relating to public spaces.

4. To identify potentially modifiable environmental factors to reduce the potency of safety discourses and increase independent mobility and physical activity for children.

5. To objectively measure the relationship between children's independent and dependent mobility and physical activity.

6. To investigate associations between neighborhood contextual factors and children's independent mobility and physical activity.

7. To contribute children's voices in local planning for city growth management.

## Methods/Design

### Study design

**Figure 1:**
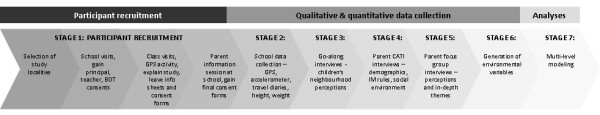
**Diagram of the stages taken to participant recruitment and data collection in the Kids in the City study**. Notes: BOT = School Board of Trustees, CATI = Computer Aided Telephone Interview, GPS = Global Positioning System, IM = independent mobility.

The KITC study used a mixed methods approach, combining a cross-sectional multi-level (child, family, school neighborhood) design to investigate associations between neighborhood environment attributes, and children's independent mobility and physical activity, with 'go along' interviews with children (researchers accompanying children on a neighborhood walk) and focus group discussions with parents to explore perceptions and experiences of their neighborhood. Figure 1 shows the stages taken to achieve participant recruitment and data collection. Pilot work for this research was completed in November-December 2010 [30,31], at which time school recruitment for the main study also commenced. Data for the main study were collected between March and June 2011. Ethical approval to conduct the research was provided by the three universities involved (AUTEC: 10/208, 18 Oct 2010; MUHECN: 10/053, 16 Aug 2010; UoA: 15 Oct 2010). Overall, 160 children aged 9-11 years and their parents/caregivers participated in the main study. The sample size estimate was derived by generating data for six hypothetical neighborhoods using New Zealand accelerometer data and defining a significant neighborhood physical activity intensity effect of at least 1.6 times greater in the neighborhood with the highest compared with the lowest physical activity intensity value. Anticipating a maximum of 20% data attrition due to faulty equipment, non-compliance, and participant dropouts, at least 25 children had to be recruited per school, with a maximum of 30 children (limited by equipment availability).

### Stage 1: Participant recruitment

#### School selection and consent

Selection of study localities was undertaken in consultation with local government and Housing New Zealand Corporation ((HNZC); government housing provider). Recognizing the potential influence of walkability (an index combining measures of street connectivity, dwelling density, land use mix and retail floor ratio) [32] and access to services and amenities (an index of walking access to child-appropriate destinations) [33] on physical activity and independent mobility, Auckland maps of walkability and destination access were also referenced in locality selection. Potential schools for recruitment were then identified using a strategy of pairing schools with a similar school decile rating (an indicator of socio-economic status of a school's catchment area), and differing neighborhood walkability and neighborhood access scores. Contact was made with the Principals of seven potential schools and six agreed to participate. Table 1 provides basic characteristics of schools involved in the study. On school selection and Principal and Board of Trustee consent, class teachers were approached, the study explained to them, and their consent sought for the children to be involved in the research.

**Table 1:** Characteristics of participating schools and areas

Region	Town	School	Decile	Walkability	Roll	% Māori, Pacific, European, Asian/South Asian, Other
South	Manurewa	Clendon Park	1	Low	441	51, 41, 1, 0, 7
South	Wiri	Wiri School	1	Medium	470	39, 57, 0, 0, 4
West	Avondale	New Windsor	5	Low	540	15, 19, 19, 27, 10
West	New Lynn	New Lynn	4	High	297	21, 34, 15, 24, 6
East	Glen Innes	Point England	1	High	448	30, 63, 4, 0, 0
East	Mt Wellington	Panama Rd	1	Low	289	21, 64, 0, 0, 14

Note: Walkability was assessed using a combination of street connectivity, residential density, land use mix, and retail floor area ratio as described in-text

#### Classroom activity

A preliminary classroom-based session was conducted with each participating class to introduce members of the research team, explain the study process, show students the research equipment, explain how Global Positioning System (GPS) units work, and give children the opportunity to use the equipment. The main aim of this process was to engage the children with the study and allow them to develop rapport with the research team. Child and parent/caregiver (hereafter parent) information sheets and consent forms were left with the teacher for distribution.

#### Parent information sessions

In addition to the classroom activity, parents were invited to attend an information session to have the study process explained to them in more detail. Sessions were held at the school at a time convenient to parents (usually early evening on school days). Members of the research team presented the research process and time was allocated for parents to have their questions answered.

#### Participant consent

There were multiple stages to the consent process; parent consent and child assent were required for the child to participate in the school-based data collection and go-along interviews; separate parent consent was required to participate in the individual telephone interview and focus groups.

### Stage 2: School data collection

**Figure 2:**
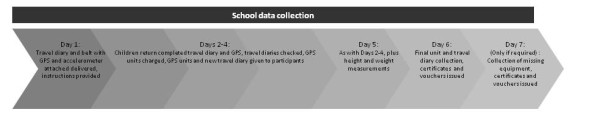
**Overview of processes taken on school visit days**. Note: GPS = Global Positioning System.

Researchers visited child participants at their school for six consecutive weekdays to collect objective physical activity and independent mobility data, travel diary data, and to charge the GPS units. In each school, a research 'office' was set up where the researchers met the children, distributed equipment, and downloaded data. Figure 2 outlines the processes undertaken on each school visit day. On Day 1, participants were fitted with a belt with a GPS unit and accelerometer attached and provided with verbal and written instructions on belt wear and removal. Participants were also provided with a colored rubber wrist watch (for recording trip times) and a travel diary for that evening and the following morning. For the next four school days, the children met with the researchers each morning to return the GPS unit and completed travel diaries (the accelerometer and belt were not taken off during this time). GPS units were put on to charge immediately by the researchers and travel diaries were checked with the children before they returned to class. GPS data were downloaded and the units reset by researchers. After lunch, the charged reset GPS units and travel diaries were returned to the children. On Friday afternoons, children were also provided with a GPS charger to take home and verbal and written instructions regarding charging the GPS unit each night. Participant incentives included stickers on completion of travel diary information and return of equipment and a shopping centre voucher offered at the end of data collection.

#### Physical activity

Physical activity was measured objectively for seven consecutive days using Actical accelerometers (BMedical Pty Ltd, Milton, Queensland, Australia) fitted to a purpose-built Actical neoprene waistband. A seven-day continuous monitoring period is recommended to gather a reliable estimate of children's activity levels, and to enable the comparison of physical activity levels on weekend and week days [34]. The Actical unit is a small (28 × 27 × 10 mm) waterproof accelerometer that is capable of measuring omni-directional movement at a sampling rate of 32 Hz; these accelerometers have been validated for measuring activity-related energy expenditure in children using indirect calorimetry [35]. Actical accelerometer count thresholds have subsequently been validated using calorimetry to classify time spent sedentary, and in light, moderate, and vigorous intensity physical activity in children [36].

Accelerometers were tested before field work started, including 60 min of sedentary time (left on a desk), being worn for a 30 min walk, and being left recording data recording overnight. All units recorded data appropriately and were utilized for data collection. Prior to distribution at each school, accelerometers were prepared to collect data in 30 s epochs and record step counts. While acknowledging the importance of using the shortest possible epochs to capture potentially intermittent patterns in children's physical activity [37], the Actical has a limited data storage capacity. The use of a 30 s epoch was therefore a pragmatic compromise allowing the storage of seven days of continuous data as well as step count information. Other than a participant identification number, no other participant information was added at this time. Accelerometers were distributed on Day 1 when participants were shown appropriate belt placement, and given verbal and written instructions for belt wear. Participants were requested to wear the belt and accelerometer for all waking hours over the next seven days, and to remove the belt only when bathing, sleeping, or participating in water-based activities. On Day 6 (corresponding to seven days of data collection), belts were collected from the children and accelerometer data were downloaded using Actical 2.12 software.

Accelerometer data were first visually screened for patterns that might indicate the data were corrupt, in which case they were removed. For all other data files, participant age, sex, and objectively-measured height and weight were entered into the Actical file. Wear times were determined using travel diary information and visual examination of the accelerometer count data. Where these did not agree, the accelerometer data were deemed to be the most accurate. Custom intervals for wear times were set within the Actical software and data extracted for wear times only as described previously [38]. An automated process was then used to screen for and remove outliers and hypothesized non-wear time that was not reported in the travel diaries (a conservative criterion of > 120 consecutive min of 0 counts was used to classify non-wear time [39]), and to apply physical activity intensity to the raw data using the thresholds of Puyau et al. [36] (halved due to our data being in 30 s epochs) as follows: sedentary, < 50 counts/30 s; light, 50-749 counts/30 s; moderate, 750-3249 counts/30 s; vigorous, > 3249 counts/30 s. Once cleaned, data were included for further analyses if at least 10 hours of data were gathered per day, for a minimum of 5 days.

#### Mobility

QStarz BT-Q1000 and BT-Q1000XT GPS units (Qstarz International Inc., Taiwan) were used to measure children's spatial location. Key differences between the models are greater tracking sensitivity and number of channels in the 1000XT compared with the earlier 1000 model (66 channels, tracking sensitivity of 165 dBm, and 42 h battery life *versus *51 channels, 158 dBm sensitivity, 32 h battery life). Dimensions of both models are 72.2 × 46.5 × 20 mm, they can both record data in epochs as short as 1 s, and have similar advertised accuracy (to within 3.0 m) and acquisition times (hot = 1 s, warm = 33 s, cold = 35-36 s). An investigation of reliability and accuracy across a range of commonly-used GPS units in activity monitoring has shown relatively high accuracy across a range of sites (e.g., under canopy, open sky, urban jungle) and good inter-unit reliability in the QStarz when compared with other units [40].

**Figure 3:**
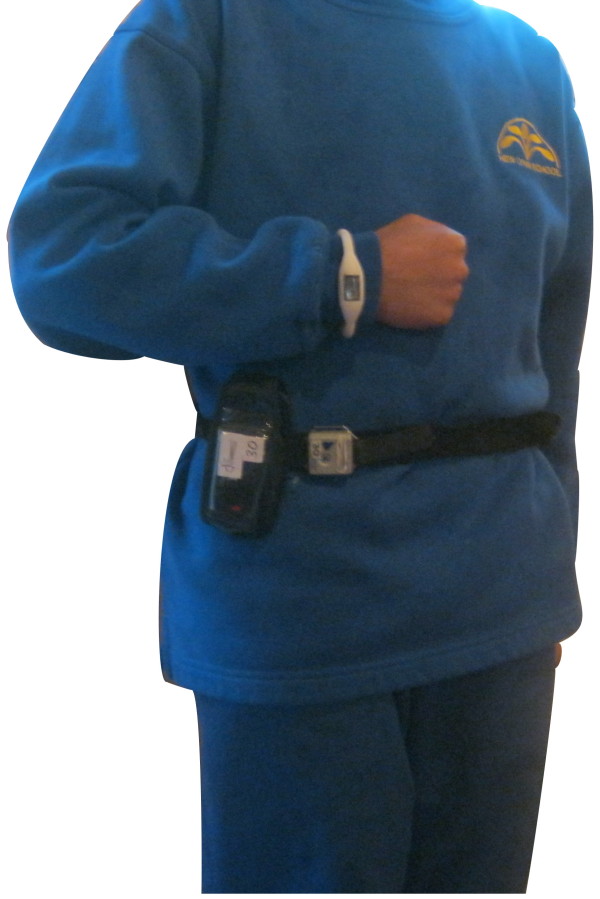
**Image of children wearing their belts with GPS unit and accelerometer attached**.

Prior to Day 1 delivery, GPS units were set up for data collection using the QStarz QTravel v1 Recorder PC utility software (QStarz International Inc., Taiwan). Units were configured to log data every 10 s, a compromise between optimal frequency (i.e., 1 s) and data storage capacity of the units over a 24 h period for weekdays (72 h for weekends). The GPS buzzer/alarm was set to off, and units were set to record: date, time, longitude latitude, height, speed, distance, dilution of precision (DOP), and satellite information (i.e. number of satellites in view, number of satellites and the signal strength of each satellite). GPS units were then turned off, labeled, and inserted in a Nokia E51 leather pouch with a clear front and clip back. Immediately prior to delivery on Day 1, GPS units were turned on to logging mode and checked for adequate satellite acquisition before being clipped to the belts given to children to wear (Figure 3). GPS data were downloaded at the schools daily using the QStarz QTravel v1 Travel Recorder PC utility software; both .kml and raw .csv data files were generated and saved. The file sizes of the GPS data files were checked to identify GPS units that were not recording data. Problematic GPS units were replaced with spares at this stage in order to minimize data loss. After the data was saved the GPS unit logs were cleared.

After data collection the .csv files were pre-processed using a custom script written in *R*. The script merges the downloaded .csv files into one file for each child, adjusts data columns so that file can be imported into Geographic Information Systems (GIS) software (i.e. renames the columns and corrects the latitude), adds data columns for different formats of date and time, discards invalid data points identified using speed, distance, DOP values, satellite information, and unit wear times, and writes the resulting dataset to a new .csv file. The .csv files were then imported into ArcGIS 9.3 (ESRI Inc., Redlands, CA) and converted into point shapefiles. Participant home and school addresses and land use data were used to assign a location type (e.g., home, school, park, shops) to each GPS datum. ET Geowizards was used to convert the GPS data points into a route for each day for each child [30].

#### Travel diary

**Figure 4:**
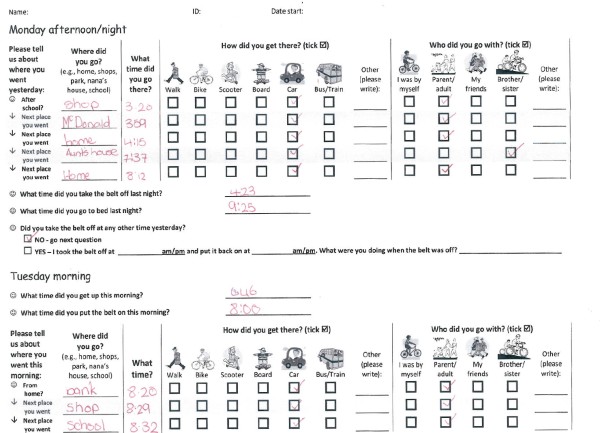
**Example of a completed travel diary for one week day, with participant identification information omitted**.

On each day of data collection, children were provided with an A4 paper travel diary to self-complete that evening and the following morning (Figure 4). Data for time, destination, mode of travel, and accompaniment for all trips taken were collected in the travel diary, as well as waking and bed times, and belt attachment and removal times. For weekend days, a full A4 sheet was provided, allowing up to eight trips per day to be recorded. Diaries were checked and confirmed each weekday with the children by a member of the research team.

#### Body size measures

Height and weight were assessed at the penultimate day of data collection at the school. Measurements were taken by one of three researchers trained to follow International Society for the Advancement of Kinanthropometry [41] and New Zealand Health Monitor Survey [42] protocols for body size measurement. Height (shoes off) was assessed to the nearest 0.1 cm using a stadiometer (Mentone Educational Centre, Victoria, Australia) and weight (in light clothing) to the nearest 0.1 kg using calibrated Seca 770 scales (Protec Solutions Ltd, Wellington, New Zealand). Measures were repeated twice; if these measures differed by more than 0.5 cm or 0.5 kg for height or weight, respectively, a third measure was taken. The final height and weight measures were determined by averaging the two closest measures. Body mass index (BMI) was calculated as weight (kg)/height (m)^2^. BMI status (i.e., normal/underweight, overweight, and obese) was determined using the age and sex-specific International Obesity Task Force thresholds [43].

### Stage 3: Go-along interviews

The go-along interview method involved the participant taking the researcher on a neighborhood walking tour, during which in-depth qualitative interviewing techniques were used to gather data regarding participant experiences, perspectives, and practices related to the environment in which they live [44]. This methodology provides a rich source of information on environmental issues from children's, rather than researchers' perspectives, by prioritizing the participant as an active agent in research.

To encourage rapport-building, ease of dialogue, and sharing of local knowledge, student interviewers were recruited to undertake the go-along interviews with the children in this study. Senior students (male and female, 16-18 years) were recruited from the local secondary school, and were matched to the sex of the child. Students were identified by the Principal of each secondary school, and on consenting to be involved with the study, were trained in qualitative interviewing procedures by members of the research team. Go along interviews started from the child's home, from where they took the student interviewer on a self-directed walk around the local neighborhood. The student interviewer carried a GPS worn on a lanyard around the neck, and a list of questions/prompts. The participant wore a digital voice recorder on a lanyard around their neck. The child was also given a digital camera to take photos of places of interest to them along the walk. For child and student interviewer safety, a member of the research team followed behind at all times during the go-along interview. Prior to starting the go-along walk, the 'following researcher' discussed the general aims, objectives, and process of the interview, checked that the child could use the camera and asked about the places the child wanted to visit and the route they wanted to take. The following researcher recorded the walking tour route on a map and took photos of the general environment, including aspects related to known or perceived desirability and safety.

Go along interview data were transcribed and analyzed thematically within NVivo (QSR, VIC, Australia) using a systematic inductive process [45,46]. Multiple readings and discussion of transcripts by more than one researcher preceded structuring a coding frame. Text excerpts were sorted into theme files and thematic descriptions developed. Children's interview data within each neighborhood were analyzed independently and once stable interpretations were concluded for each case study neighborhood the data were examined for convergent and divergent understandings by neighborhood. To the extent possible given the sample characteristics, commonalities and differences in experience and meaning between genders and ethnic groups were also examined. GPS data were downloaded as described earlier and a spatial map of the route taken generated within Google Maps. Photos were linked with the GPS route to provide spatial representation of sites of interest along the tour. A broad coding system was also applied to the photos taken (e.g., home, friend's house, shops) to enable the quantification and identification of environmental features that were important to children.

### Stage 4: Parent interviews

Parents were telephoned at the completion of data collection in schools and a 75 item computer-aided telephone interview (CATI) was administered by a trained researcher. The survey drew from existing questionnaires and included child, parent, and household demographics; perceptions of neighborhood physical and social environments [47]; social cohesion [48]; children's mode and accompaniment of trip to and from school, independent mobility to other settings, parent neighborhood safety concerns and independent mobility considerations, trip chaining; play locations; household car and bike availability; and parental perceptions of the importance of their child's independent mobility and interactions with friends. The parents were also asked about their transport to school and independent mobility as children [11]; CATI interviews lasted between 15-20 min, and were conducted in the parent's language preference of English, Samoan, Tongan, or Chinese.

### Stage 5: Focus groups

Parents were invited to participate in a focus group discussion with 6-7 other local parents. Focus group discussion centered on parental perceptions and experiences of neighborhood and the factors that influenced their decisions on when and where their children could play and travel alone and accompanied. Focus groups were held in the early evening at the school and lasted approximately 60 min. The discussion was audio taped, transcribed, and thematic induction analyses were conducted as described earlier (Stage 3: Go-along interviews).

### Stage 6: Generation of environmental factors

#### Weather

Sunlight hours, ambient temperature, and rain-free days have been previously related to objectively assessed physical activity in child populations in New Zealand [49,50]. Weather variables were therefore created to examine or control for the weather effects on engagement in daily physical activity and/or independent mobility. New Zealand weather data are collected at ten minute intervals at 6,500 stations nationwide by the National Institute of Water and Atmospheric Research Ltd and are freely available from the New Zealand National Climate Database http://cliflo.niwa.co.nz/. Raw daily frequencies for minimum and maximum temperature (°C), rainfall (mm), and sunlight hours for weather stations closest to each school were downloaded from this database and matched to the daily activity and mobility data by date and region.

#### Streetscape audit

A modified version of the Systematic Pedestrian and Cycling Environmental Scale (NZ-SPACES) was used to gain a systematic objective measure of neighborhood attributes associated with physical activity (e.g., aesthetics) that could not be captured by GIS-based tools. NZ-SPACES has demonstrated reliability in measuring attributes such as street safety, aesthetics, incivilities and pedestrian infrastructure [51,52], and has been adapted for and used previously in the New Zealand context. In each study neighborhood, all street segments falling completely within an 800 m network buffer around the school were selected for auditing. Most segments were audited virtually within Google Street View [53]. In instances where urban developments were occurring or Google Street View was unavailable or known or suspected to be outdated, manual NZ-SPACES audits were conducted instead of virtual audits. Street segments identified by the researcher as being too short (all were < 50 m) to audit were not audited. All streetscape audits were conducted by one researcher (SO). Intra-rater reliability was assessed in a random sub-sample of streets (10%). Objective environmental quality audits were also made manually using the New Zealand Public Open Spaces Tool (NZ-POST) [54]. NZ-POST has adequate reliability for auditing public open spaces, measuring activities, environmental quality, amenities available, and safety [55]. NZ-SPACES and NZ-POST data were aggregated at the neighborhood level by summed environmental domains (e.g. traffic safety, personal safety, walking/cycling infrastructure, aesthetics) and overall scores.

#### GIS variables

Massey University holds spatial databases that include existing road network information, bus/train/ferry routes and stops, parks and reserves, and a range of community facilities. The territorial authorities in the study areas provided the necessary additional information (zoning, building outline data and updated amenity data) to enable the development of GIS variables to examine the relationship between the urban neighborhood environment and children's activity and independent mobility. Specific GIS-derived variables were generated in ArcInfo 9.3 (ESRI Inc., Redlands, CA) using an 800 m street network buffer around the child's home and school including, but not limited to:

##### Neighbourhood Destination Accessibility Index (NDAI)

The NDAI is a GIS-based measure that uses eight domains of neighborhood destinations (education, transport, recreation, social and cultural, food retail, financial, health, other retail) to determine overall pedestrian accessibility to neighborhood destinations and intensity of these destinations within given boundaries [33]. NDAI scores can range from 0 to upwards of 30; differences in NDAI have been identified across New Zealand cities [33]. NDAI scores were calculated for the study region.

##### Street connectivity

Intersection density was first calculated as the ratio between the number of intersections with three or more legs to the block land area and intersections with three or more unique junctions extracted from the road network. Street connectivity was calculated as the number of intersections/square kilometer within 20 m of each mesh-block boundary. Values for each mesh-block were between 0 and 1, where a score closer to 1 indicated higher street connectivity.

##### Net residential density

Total number of occupied private dwellings was derived using mesh-block data from the New Zealand 2006 census. Residential land area was obtained from zoning data provided by the territorial authorities. Residential density was calculated as the ratio of dwellings to the residential land area for each block.

##### Land use mix

An entropy score was developed considering residential, retail, entertainment, office, and institutional land mixes. Land uses within each mesh-block were defined as commercial, residential, industrial, open space, or other using zoning data. Land use mix was calculated using an entropy index, where 0 indicates homogeneity of land use, and a value closer to 1 indicates greater heterogeneity of land uses.

##### Percentage greenspace

The percentage of the area in greenspace was calculated by dividing the area of greenspace by the area in the 800 m buffer.

##### Distance from home to school

The shortest network route to school was calculated using ArcGIS Network Analyst.

### Stage 7: Multi-level modeling

At the time of printing, this final research stage was in process; research findings will be published in subsequent papers. For the purposes of this study, a three-level model will be used to examine the relative influence of factors at the individual, family, and school/neighborhood levels on children's physical activity and independent mobility. Strengths of this approach are that it acknowledges and accommodates the clustering of child outcomes within schools/neighborhoods and households; and enables potential important confounders to be controlled for, while allowing random interactions among etiologic factors at different levels to be examined. Further, multilevel modeling facilitates a more ecological understanding of factors associated with children's behavior for better targeted interventions [56].

Associations between potential predictor factors and children's physical activity and independent mobility will be examined using generalized estimating equations (GEE). GEEs allow the relationship between repeated measures of physical activity and independent mobility to be analyzed simultaneously and corrects for within-subject correlations. Moreover, GEE models handle unbalanced and data missing at random [57]. Neighborhood variables important in measuring children's level of mobility and physical activity will be determined using univariable GEE modeling. Multilevel modeling will then be used to investigate associations between neighborhood and household level variables and outcome variables whilst controlling for basic child and household demographic data, weather, and other factors of importance. Household demographic data may be treated as predictor or outcome data depending on the research questions.

## Discussion

Children's independent mobility and physical activity have been declining over recent decades and it is unclear whether the planned residential intensification of urban neighborhoods will diminish or exacerbate this trend. How children fare in intensifying cities and the implications of residential intensification for the independent mobility and physical activity of resident children is largely unexamined [9,29]. Higher density developments are seldom planned with children in mind and commonplace discourse in New Zealand and elsewhere attests these settings are largely unsuitable for families [58-62]. With 87% of New Zealand's population living in urban settings, understanding how urban neighborhoods inhibit or promote children's independent mobility and physical activity is vital for children's current and future health and wellbeing. Neighborhood design has the potential to increase children's everyday mobility with beneficial impacts on energy expenditure, body size, and children's agency. Developments that fail to promote children's physical activity will be neither environmentally nor socially sustainable in the long term and may contribute to increasing health disparities [14]. This research is a unique and timely opportunity to bring a children's health and wellbeing perspective to the planning process. Research findings will inform future research and interventions for facilitating physical activity and independent mobility for children, and will contribute to the theoretical evidence base of multilevel modeling of environments and health [63].

As a cross-sectional study, research findings cannot be used to determine causality, and the recruitment strategy utilized limits generalizability of research findings to children residing in more socio-economically disadvantaged, higher residential density, urban areas. The multidisciplinary nature and ecological approach taken are key strengths of the study. In keeping with the Child Friendly Cities program [64] and others [65] calling for greater participation by young people in urban planning, this research considers children as active agents in the research process. Participatory GIS techniques and go-along interviews are utilized as enabling communication tools to elicit children's environmental perspectives in their own words [66]. Acknowledging the influence of the family environment and the fundamental role of parents as gatekeepers to children's activity behaviors, parental factors are gathered using in-depth qualitative and quantitative survey and focus group methodologies. Objective quantitative data on children's independent mobility and physical activity are collected using current best practice epidemiological approaches. Advancing on earlier research [67,68], objective measurement of children's independently mobile trips have been quantified in detail, drawing from travel diary, GPS, and accelerometer data. Built environmental features have been objectively quantified utilizing a variety of tools, providing a detailed quantification of this factor in relation to children's activity and mobility. Importantly, engagement with territorial authorities and related government agencies (e.g., HNZC) in the development of this research provides effective pathways for the strategic translation of research-based evidence. Local government has a key role in providing safe and accessible environments, which includes local streets as well as amenities for children. Through these existing channels an important opportunity exists to directly inform neighborhood development and renewal in specific places, and enable the "voice" of children to contribute to community-council dialogue on neighborhood planning and development.

## Abbreviations

AUTEC: Auckland University of Technology Ethics Committee; BMI: Body mass index; °C: Degrees Celsius; CATI: Computer Aided Telephone Interview; DOP: dilution of precision; GEE: Generalized Estimating Equation; GIS: Geographical information systems; GPS: Global Positioning System; h: height; HNZC: Housing New Zealand Corporation; KITC: Kids in the City study; m: meters; min: minutes; mm: millimeters; MUHECN; Massey University Human Ethics Committee; kg: kilograms; NDAI: Neighbourhood Destination Accessibility Index; NZ-POST: New Zealand Public Open Spaces Tool; New Zealand NZ-SPACES: New Zealand Systematic Pedestrian and Cycling Environment Scan; s: seconds; UoA: University of Auckland.

## Competing interests

The authors declare that they have no competing interests.

## Authors' contributions

MO developed the first draft of the manuscript. KW, RAK, SM, HMB, PC, LA, PS, and HB contributed to the conception and the design of the study. All authors provided critical feedback during manuscript development. Each author has read and approved the final manuscript.

## Pre-publication history

The pre-publication history for this paper can be accessed here:

http://www.biomedcentral.com/1471-2458/11/587/prepub
